# Narrow and Stripe Leaf 2 Regulates Leaf Width by Modulating Cell Cycle Progression in Rice

**DOI:** 10.1186/s12284-023-00634-3

**Published:** 2023-04-18

**Authors:** Wenqiang Shen, Jiajie Sun, Zan Xiao, Ping Feng, Ting Zhang, Guanghua He, Xianchun Sang

**Affiliations:** Rice Research Institute, Key Laboratory of Application and Safety Control of Genetically Modified Crops, Engineering Research Center of South Upland Agriculture, Ministry of Education, Academy of Agricultural Sciences, Southwest University, Chongqing, 400715 China

**Keywords:** Rice, Narrow leaf, Ribonucleotide reductases (RNRs), Cell cycle

## Abstract

**Background:**

Leaf morphology is an important component of the idea plant architecture that extensively influences photosynthesis, transpiration, and ultimately grain yield in crops. However, the genetic and molecular mechanisms regulating this morphology remain largely unclear.

**Results:**

In this study, a mutant showing a narrow and stripe leaf phonotype, designated nsl2, was obtained. Histological analysis revealed defects in the vascular system and reduced epidermal cell number in the *nsl2*, while the cell size remained unchanged. Map-based cloning and genetic complementation experiments revealed that *NSL2*, which encodes a small subunit of ribonucleotide reductases (RNRs), is a null allelic with *ST1* and *SDL*. The *NSL2* was expressed in variety of tissues, with the highest levels detected in leaves, and its protein was localized in the nucleus and cytoplasm. The dNTPs level was altered in the *nsl2* mutant, and thereby affecting the dNTPs pool balance. In addition, flow cytometric analysis and the altered transcript level of genes related to cell cycle indicated that *NSL2* affects cell cycle progression.

**Conclusions:**

Our findings here suggest that *NSL2* function in the synthesis of dNTP, the deficient of which leads to DNA synthesis block and in turn affects cell cycle progression, and ultimately decreased cell number and narrow leaf in the *nsl2* plant.

**Supplementary Information:**

The online version contains supplementary material available at 10.1186/s12284-023-00634-3.

## Background

Rice (*Oryza sativa* L.) is one of the most important food crops in the world, and its yield relates to food security. In rice, leaves are the main organ for photosynthesis and respiration, and leaf morphology and structure directly affect the growth, development and the grain yield (Yuan 1997). Therefore, elucidation of the molecular mechanisms of regulating leaf morphology is important for understanding the process of leaf development. A ‘super-high’ grain yield may be achieved by controlling plant architecture, and therefore this biological aspect has attracted rice breeders’ attention.

Leaf is an important organ in plant growth, and its development involves complexed processes, mainly through a series of polar development and cell differentiation (Micol and Hake 2003). Leaf formation develops from the shoot apical meristem (SAM). Cells in the peripheral zone at the flank of the SAM are transformed into leaf primordia. Leaf initiation is followed by the establishment of polarity, which includes proximo-distal axis in the longitudinal, adaxial–abaxial axis in the up-down direction, and central–lateral axis in the middle-to-side direction (Moon and Hake 2011). Subsequently, leaf primordium transforms into mature leaf through cell expansion and division (Gonzalez et al. 2012). Therefore, the complexed coordination of cell expansion and division controls leaf morphology, and the number of leaf veins is an obvious characteristic of leaf blade width mutants. From previous reports, the genes that regulate leaf morphology can be mainly divided into three types: transcription factors, hormones, and micro RNA. Studies have shown that WUSCHEL-related homeobox (WOX) gene demarcates the middle region of leaf primordium, forming the center that organizes lateral leaf growth and marginal development (Nakata et al. 2012; Wang et al. 2020). *NAL2* and *NAL3* belongs to the WOX3A family, which are orthologous to duplicate genes *NS1* and *NS2*, and *PR* in Arabidopsis. *NAL2* and *NAL3* play important roles in lateral-axis outgrowth and vascular patterning in leaves. *nal2/3* developed a narrowed leaf, caused by a reduced number of vascular bundles and the lack of lateral domain (Ishiwata et al. 2013).

As a major plant hormone, auxin plays an important role in leaf development, precisely regulating leaf primordium differentiation and cell growth. *NAL1* encoding a plant-specific protein and mainly expressed in vascular tissues. A mutation of this gene leaded to a significant decrease in the polar transport activity of auxin, which changed the polar transport of auxin, and reduced in the leaf blade width and number of longitudinal veins (Qi et al. 2008; Cho et al. 2014). Furthermore, *NAL1* regulates cell division as early as during leaf primordia initiation, affecting the transcript levels of G1-and-S-phase-specific genes (Jiang et al. 2015). *NAL7* encodes a flavin-containing monooxygenase, which is a member of the YUCCA family and regulates leaf width by participating in auxin biosynthesis (Fujino et al. 2008). *TDD1* encodes a protein homologous to anthranilate synthase β-subunit, involved in Trp-dependent auxin biosynthesis. The contents of tryptophan and auxin in embryos and flowers of *tdd1*decreased significantly, indicating that the mutant phenotypes of *tdd1* were caused predominantly by Trp and auxin deficiency (Sazuka et al. 2009).

MicroRNAs are known to be involved in developmental regulation, genome maintenance, and defense in eukaryotes. The *OsDCL1* gene cleaves microRNA from the precursors during microRNA processing, loss of function of *OsDCL1* results in abnormal phenotypes such as narrow leaf, dwarfism and short root (Liu et al. 2005). Interestingly, *OsDCL1* not only plays an important role in microRNA processing, but also functions in the immunity to rice blast fungi (Zhang et al. 2015). Additional genes are involved in leaf development. *NRL1* encodes the cellulose synthase-like protein D4 (OsCslD4). A mutation of the *NRL1* results in a decreased number of longitudinal veins in leaves, producing phenotypes with reduced leaf width (Hu et al. 2010; Wu et al. 2010). *AVB* encoding a novel protein with unknown function, has been found to be involved in the maintenance of the normal cell division pattern in lateral primordia development and procambium establishment, associated with auxin signaling (Ma et al. 2017).

Ribonucleotide reductases (RNRs) catalyze the conversion of ribonucleotides to deoxyribonucleotides, contributing to a rate-limiting step in DNA precursor replication (Guarino et al. 2014). Eukaryotic RNR is a heterodimers composed of two large subunits, R1, and two small subunits, R2 (Jordan and Reichard 1998; Nordlund and Reichard 2006). In *Arabidopsis thaliana*, *CSL8* and *TSO2* encode the large subunit and the small subunit of RNR, respectively, the mutants showed bleached leaves and siliques (Wang and Liu 2006; Garton et al. 2007). Furthermore, the number of chloroplast in *cls8* reduced, and the chloroplasts were dysplastic (Garton et al. 2007). In rice, the mutant *v3* and *st1* with disrupted the large and small subunit gene of RNR, respectively, and are growth stage-dependent and environment-dependent leaf chlorosis (Yoo et al. 2009). Similarly, *STL1* encodes the large subunit of RNR in *S. viridis*, *stl1* showed growth retardation as well as a rolled and bleached leaf. Additionally, chloroplast biogenesis was suppressed due to presence of undifferentiated chloroplasts in *stl1* (Li et al. 2022). It has been proved that RNR plays an important role in regulating leaf color. However, the molecular mechanism that RNR regulates leaf width has not been fully elucidated.

Here, we identified a *narrow-stripe leaf 2*(*nsl2*) mutant, generated by ethyl methane sulfonate (EMS) mutagenesis, which showed narrow and stripe leaf phenotype. Map-based cloning revealed that *NSL2* encodes the small subunits of ribonucleotide reductase (RNRS1), which is allelic to the previously identified loci *STRIPE 1* (*ST1*) and *STRIPE AND DROOPING LEAF* (*SDL*) (Yoo et al. 2009; Qin et al. 2017). Although a function of RNRS1 in leaf color and drooping has been reported, the role of RNRS1 in controlling leaf width has not been studied in detail. Subcellular localization analysis demonstrated that NSL2 was localized in the nucleus and cytoplasm. *NSL2* plays a critical role in the expression of genes associated with cell cycle. These findings demonstrate the important role of *NSL2* in regulation leaf morphology, which provided a novel perspective on the functional study of *RNRS1*.

## Results

### Characterization of *nsl2* Mutant

**Fig. 1:**
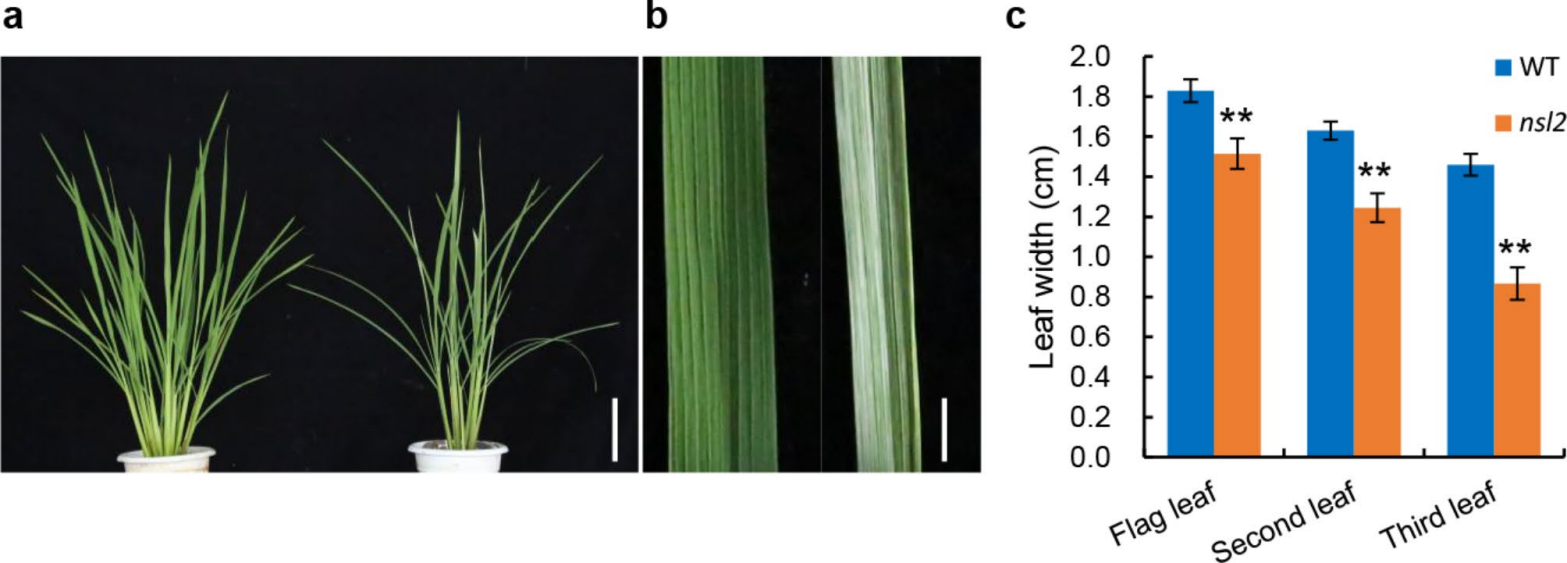
Phenotypic characteristics of the *nsl2* mutant. **a**: Phenotypes of the WT (left) and *nsl2* (right) at the tillering stage. **b**: Differences of leaf shape and leaf color in the adaxial-side between WT (left) and *nsl2* (right). **c**: Comparison leaf width of the upper three leaves between WT and *nsl2*. Bars (**a**) 10 cm; (**b**) 1 cm

**Fig. 2:**
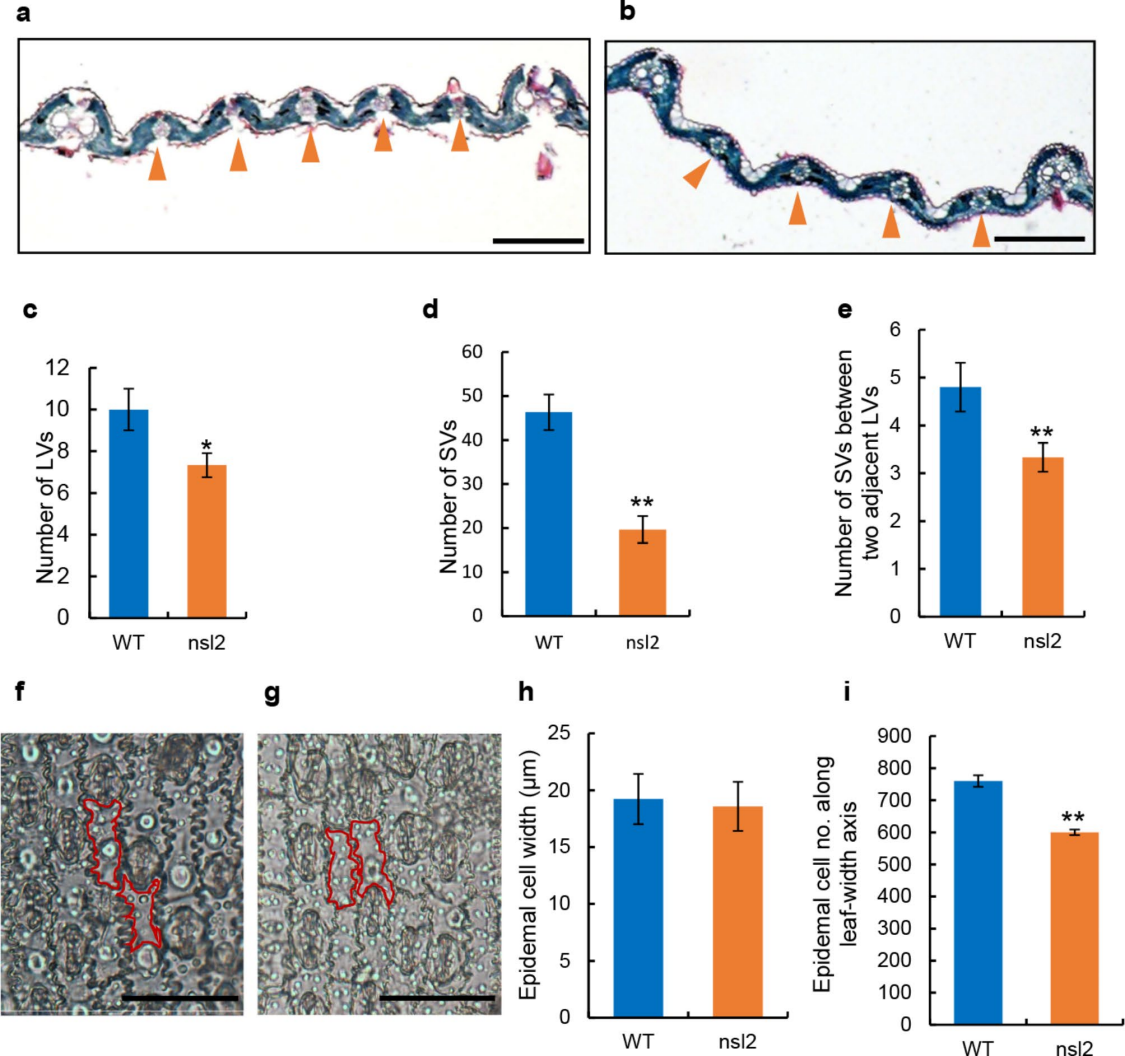
Histological analysis of WT and the *nsl2* mutant. **a and b**: Transverse sections of the expanded leaves of WT (a) and *nsl2* (b). **c-e**: Comparison of the number of large vascular bundles (LVs) (c) and small vascular bundles (SVs) (d), the number of SVs between two adjacent LVs (e). **f and g**: The abaxial epidermis of leaf blade of WT (f) and *nsl2* (g). **h and i**: Comparison of the epidermal cell width (h), and the epidermal cell number along leaf-width axis (i) of WT and *nsl2*. Bars (a-b) 200 μm; (f-g) 50 μm. Values are means ± SD (n = 10 leaves). The P-values were determined using Student’s test. *P < 0.05, **P < 0.01

The narrow-stripe leaf 2 mutant *nsl2* was successfully separated from a rice mutant population generated by EMS-induction of the indica restorer variety Jinhui 10 (J10). The plants of *nsl2* mutant showed a narrow and stripe leaf (Fig. 1a,b). Phenotypic observation and statistics of leaf growth showed that the leaf blade width of wild type (WT) at heading stage were 1.83, 1.63, and 1.46 cm, respectively. However, those of *nsl2* were 1.51, 1.24 and 0.86 cm, which attained approximately reduction of 17.5, 23.9, and 41.1%, respectively (Fig. 1c). In addition, no significant difference was found in the leaf length (Fig. S1). Vascular bundle patterning, and the cell number and size are the main cytological factors controlling leaf shape (Qi et al. 2008; Li et al. 2013). To explore the cause of the reduced leaf width, the morphological and histological observation of the middle parts of the leaves was examined at the booting stage (Fig. 2a,b; Fig. S2a,b). It was found that, compared with the WT, the average number of large and small veins in *nsl2* was reduced by 27 and 57.6%, respectively (Fig. 2c,d), and the small veins between two adjacent large veins was significantly reduced from 4.8 in the WT to 3.3 in the *nsl2* (Fig. 2e). We further studied the relationship between narrow leaf and leaf epidermal cell number and size using the microscopic approach. A significant reduction in cell number of cells along the leaf-width axis was found in *nsl2*, while the cell width was unchanged (Fig. 2f-i). These observations suggested that the reduced leaf width may have been caused by the reduced number of cells along the leaf-width axis.

Previous study has shown that *nsl2* displayed variegated and stripe leaf (sang et al., 2010). In accordance with the stripe leaf phenotype of the *nsl2* mutant, the leaf chlorophyll content was significantly reduced in the mutant compared with the WT at the tillering stages (Fig. S3). Aside from the leaf phenotype, *NSL2* also have some other effects on plant development. At the mature stage, the height of *nsl2* was significantly reduced. Compared to WT, the mature panicle traits of *nsl2*, including number of primary branches per panicle, number of secondary branches per panicle, the number of spikelets per panicle, and seed setting rate, declined markedly. However, no significant difference was found in the number of effective panicle (Table. S2). Therefore, it was concluded that *NSL2* not only affects leaf phenotype, but also has regulatory roles in panicle traits.

### Map-Based Cloning of *NSL2*

**Fig. 3:**
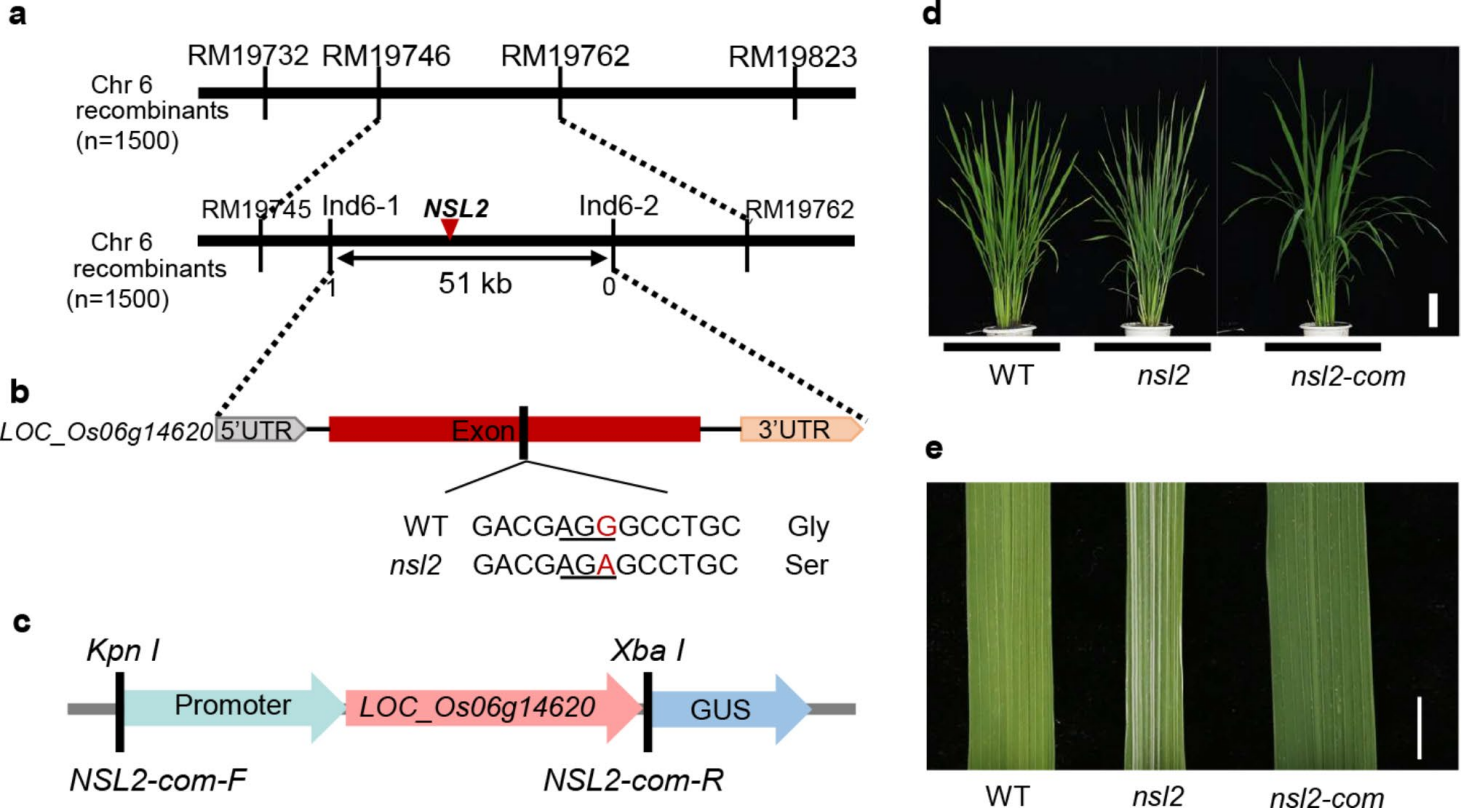
Map-based cloning of *NSL2*. **a**: Primary and fine mapping of the *NSL2* gene on chromosome 6. The *NSL2* locus was narrowed to a 51-kb region. **b**: The structure of candidate gene and mutation site. **c**: Structure of the NSL2 complementary vector. **d and e**: Phenotypes of the *NSL2* gDNA complementation lines. Bars (**d**) 10 cm; (**e**) 1 cm

The *nsl2* mutant phenotype was controlled by a single recessive gene, and *NSL2* was previously mapped to a 345 kb region between SSR marker RM19746 and RM19762 on the short arm of chromosome 6. In the current study, to fine-map and clone the *NSL2* gene further, two new InDel marker Ind6-1 and Ind6-2 and a total of 1,500 recessive plants were obtained and applied. Finally, the *NSL2* gene has been mapped in a region of 51 kb between Ind6-1 and Ind6-2 (Fig. 3a).Ten annotated genes are predicted in the mapped region (http://www.gramene.org/) (Table. S3). Sequence analysis identified a single-nucleotide substitution from G to A within the *LOC_Os06g14620* gene, which caused an amino acid mutation of Gly-212 to Ser-212 in the *nsl2* mutant (Fig. 3b). To confirm that the mutation of *LOC_Os06g14620* is responsible for the phenotypic alteration in the *nsl2* mutant, a complementary vector that contained the 2,000-bp upstream sequence, 1,020-bp coding sequence, and 899-bp downstream sequence was transformed into the *nsl2* mutant (Fig. 3c). In total, 16 transgenic lines were obtained, in 12 of which the mutant phenotypes were rescued, and the substitution site were heterozygous (G/A) (Fig. 3d, e; Fig. S4). These results finally confirmed that *LOC_Os06g14620* gene was the target gene *NSL2*. Moreover, *NSL2* is also the allele of *ST1*, *SDL* (Yoo et al. 2009; Qin et al. 2017).

### Spatiotemporal Expression Pattern and Subcellular Localization of NSL2

**Fig. 4:**
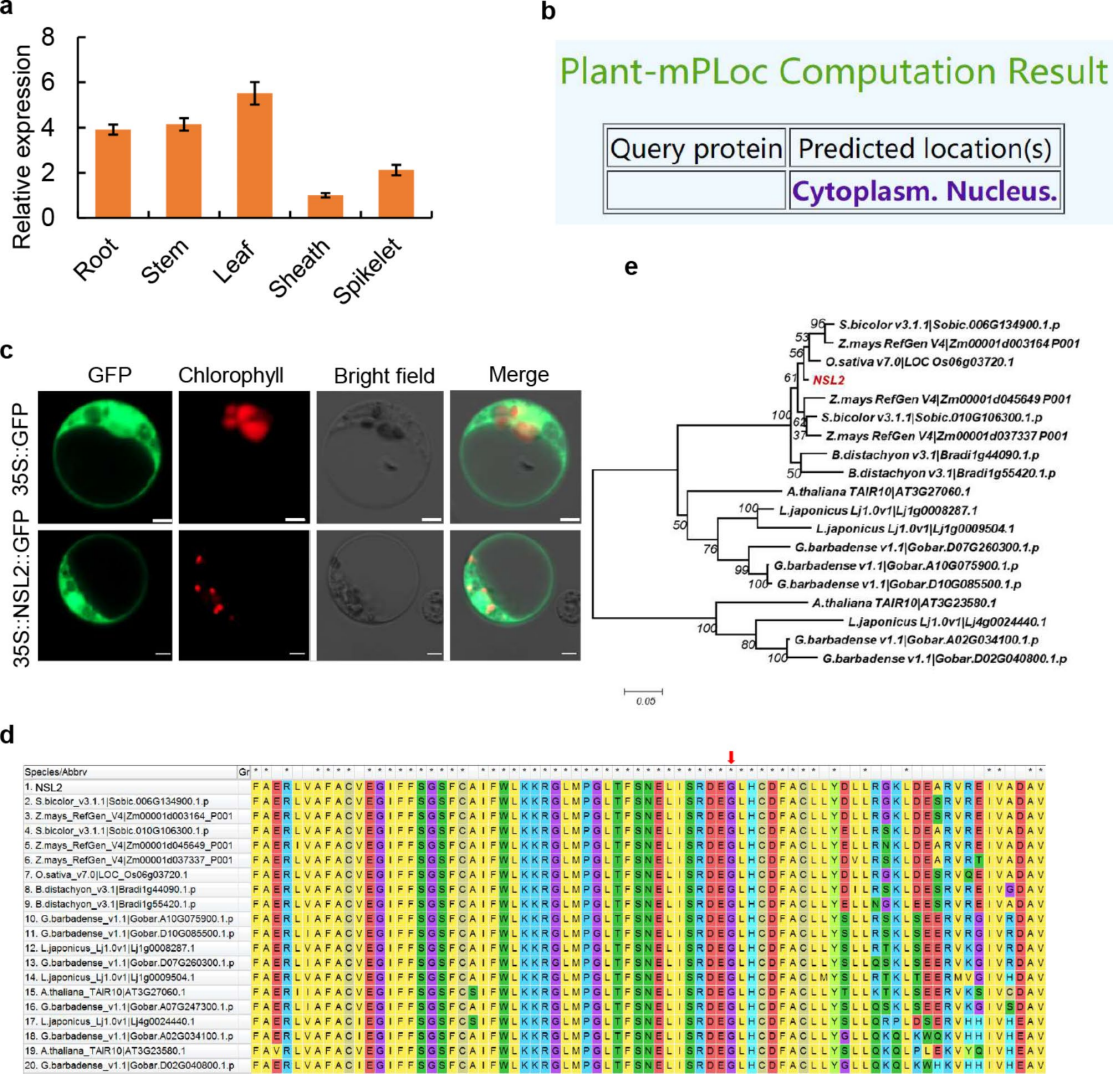
Expression pattern and protein analysis of NSL2. **a**: RT-qPCR analysis of *NSL2* expression in different tissues of WT normalized to actin. Bars represent mean of three biological repeats ± SD. **b**: Computation result of NSL2 protein. Using the Plant-mPLoc server (http://www.csbio.sjtu.edu.cn/bioinf/plant-multi/ ) **c**: Subcellular localization of NSL2-GFP in the transgenic rice leaf protoplasts. **d**: The conservation of the NSL2 protein sequence analyzed by MEGA 5.1.(The red arrow indicates the amino acid residue mutated in mutant). **e**: Phylogenetic tree of the NSL2 protein. Bars (**c**) 5 μm

To determine the spatiotemporal expression pattern of *NSL2*, quantitative reverse transcription-PCR (qRT-PCR) analysis was performed. The qPCR analysis showed that *NSL2* expressed in a variety of tissues, including the roots, stems, leaves, leaf sheaths, and panicles. The highest level of expression was observed in the leaf blade (Fig. 4a), consistent with its role in influencing leaf development.

Using the Plant-mPLoc server (http://www.csbio.sjtu.edu.cn/bioinf/plant-multi/), it was predicted that the NSL2 protein located to the nucleus and cytoplasm (Fig. 4b). To determine the subcellular localization of NSL2 protein, vectors for the NSL2-GFP fusion protein and the single GFP protein were transiently expressed in rice protoplasts. In protoplasts that expressed GFP alone, green florescence was observed throughout the cell expect in the vacuole. Green fluorescence, reflecting the NSL2-GFP fusion protein, was detected in the nucleus and cytoplasm (Fig. 4c), which was in accordance with the predicted localization of Plant-mPLoc server. These results demonstrated that NSL2 was localized in the nucleus and cytoplasm.

### NSL2 Encodes a Small Subunits of Ribonucleotide Reductase

A blastp analysis was performed on the NCBI protein database using the NSL2 protein sequence. BLAST analysis showed that NSL2 belongs to ferritin-like superfamily and *NSL2* encodes a small subunits of ribonucleotide reductase, which was previously designated *ST1* and *SDL* (Yoo et al. 2009; Qin et al. 2017), and characterized to be involved in nucleotide transport and metabolism. Multiple sequence alignment was performed by Cluster W software showed that the *nsl2* mutation site was within a conserved site (Fig. 4d).

A phylogenetic tree for NSL2-like orthologs was constructed, containing those from eudicots and monocots. The phylogenetic tree indicted that NSL2 is conserved in monocots and dicots. Notably, the *LOC_Os06g03720* that is predicted to encode RNRS2, had a high homology and close branching relationship with NSL2. In addition, putative NSL2 orthlolgs were also identified from the genomes of maize, sorghum, brachypodium distachyon, arabidopsis, cotton (Fig. 4e). The existence of NSL2 -like orthlolgs in different plant species might suggest conserved biochemical function of the NSL2 protein family.

### The dNTP Level is Altered in *nsl2*

**Fig. 5:**
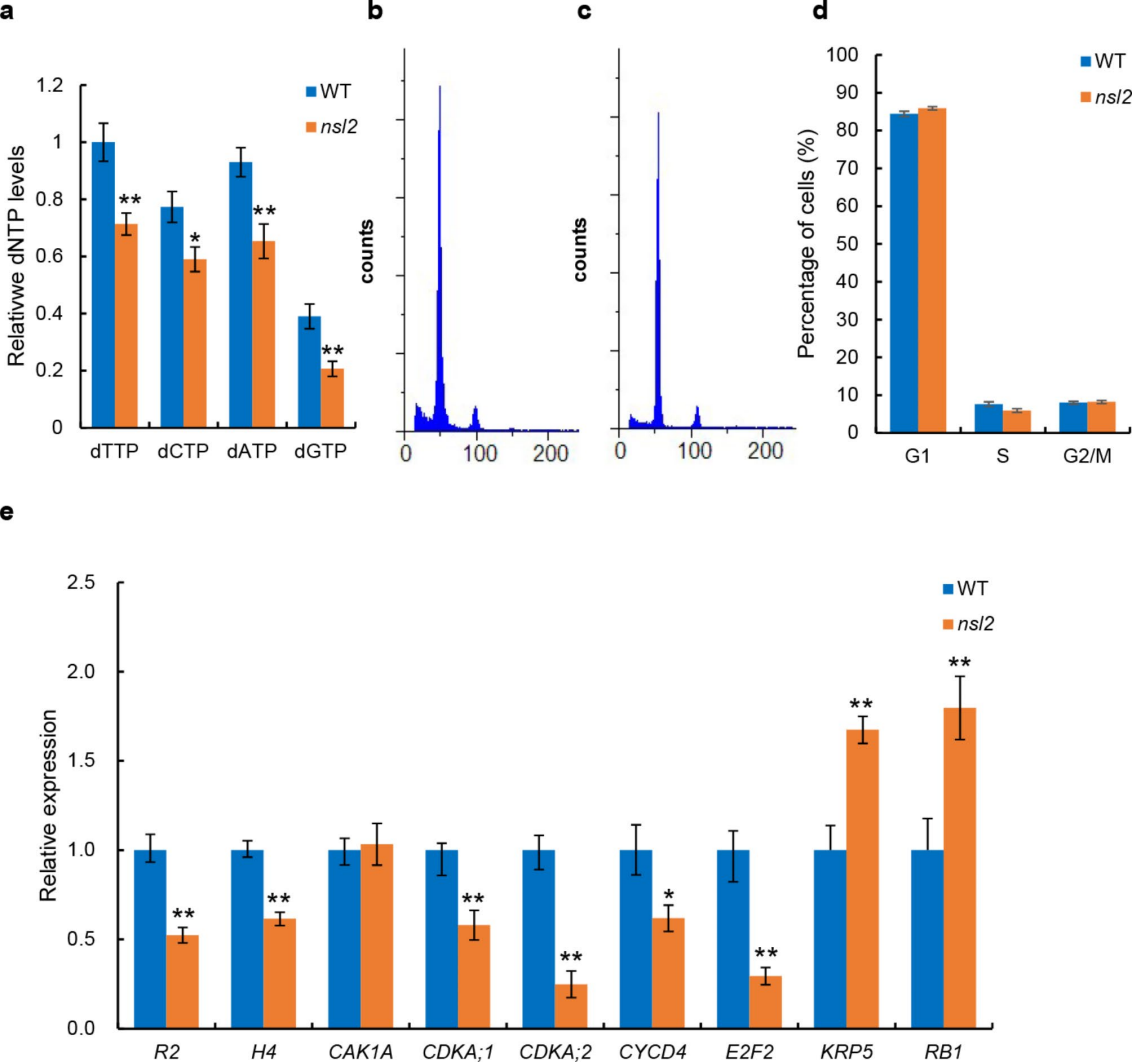
Involvement of *NSL2* in cell division in leaves. **a**: Relative dNTP levels in wild-type and *nsl2* leaves. The values were normalized to the wild-type (WT) dNTP levels. Error bars, ±SD (*n* = 3). **b and c**: Flow cytometry measurements of nuclei from the WT (b) and *nsl2* plants (c). **d**: Quantification of the DNA profiles of WT and *nsl2* plants. **e**: RT-qPCR analysis the transcript levels of cell division related genes in leaves of WT and *nsl2* plants. The P-values were determined using Student’s test. *P < 0.05, **P < 0.01

As *NSL2* encodes a small subunits of ribonucleotide reductase, which functions in the *de novo* synthesis of dNTPs, are required for DNA duplication and cell cycle progression (Wang and Liu 2006; Tang et al. 2019). We speculated the *NSL2* mutation might affect cell division due to an insufficient and/or unbalanced supply of dNTPs. To demonstrate this possibility, we first measured the dNTP levels in WT and *nsl2* using a polymerase-based method as described previously (Roy et al. 1999; Wang and Liu 2006). As expected, the levels of dNTP were shown to be reduced in *nsl2*, compared with the WT (Fig. 5a). This result suggested that the *NSL2* mutation causes dNTP insufficient supply.

### Cell-cycle Progression is Affected in *nsl2*

A high concentration of dNTPs is needed in the S-phase of cell cycle where the DNA duplication takes place (Chabes et al. 2003). To investigate whether the reduced dNTPs affects the cell-cycle progression, flow cytometry was used to monitor the amount of DNA in the nuclear suspensions in the shoot apex of WT and *nsl2* plants. The results revealed a significant decrease in the number of cells in the S phase of cell cycle (Fig. 5b-d), implying that the *nsl2* mutant had a lower cell division activity than those of WT, leading to decreased cell proliferation. This finding is consistent with the observation of the reduced number of epidermal cell along leaf-width axis. In addition, we also examined the expression levels of cell-cycle related genes. Expression level of the cyclin-dependent kinase-activating *R2* which regulates S-phase progression was down-regulated. Similarly, the S-phase marker gene *histone H4* and three G1/S-phase cell cycle marker genes *CYCD4*, *CDKA;1*, *CDKA;2*, *E2F2* were also down-regulated in *nsl2* mutant. In contrast, the CDK inhibitor, *KRP5* and *RB1*, which negatively regulate cell division, were up-regulated, compared to WT (Fig. 5e). Therefore, these results showed that the decreased number of cells in *nsl2* was caused by altered cell-cycle progression. These results concluded that the DNA duplication and progress of cell cycle in *nsl2* are indeed affected.

## Discussion

### *NSL2* Encoded a Novel Allelic Gene of *ST1* and *SDL*

In the present study, we found that a ferritin-like superfamily gene, *NSL2*, plays an important role in controlling leaf width and color. We report that the *nsl2* phenotype was induced by a mutation in the *ST1* and *SDL*, which were shown to encode the RNRS1. It was demonstrated that *ST1* regulates leaf color, *SDL* show stripe and drooping leaf, dwarfism and the abnormal floral organs (Yoo et al. 2009; Qin et al. 2017). In the *st1* mutant, a single-base change from A to G at the 119th bp which caused a missense mutation; in the *sdl* mutant, a 138-bp of repetitive sequence insert at the 475th bp of the exon. In the present study, the sequence analysis revealed that the *NSL2* gene contained a single base transversion from G to A at the 634th bp in the exon. Similar to *st1* and *sdl*, *nsl2* also exhibits stripe leaf. However, their stripes vary in degree. In the *st1* and *sdl* mutant, the cells of chlorotic leaf were found to contain fewer and smaller undifferentiated chloroplasts, in addition, the grana lamellas could not be found on chloroplast (Yoo et al. 2009; Qin et al. 2017); however, in our previous study, some irregular grana lamellas could be found in *nsl2* chloroplast (Sang et al. 2010). The destruction of chloroplasts directly affects photosynthesis which in turn affects the main agronomic traits. As a result, most of the main agronomic traits were significantly reduced in *sdl* and *nsl2*. However, no significant difference was found in the number of effective panicle and 1,000-grain weight in the *nsl2*. Additionally, histocytology revealed that the number of vascular bundles decreased in *sdl* and *nsl2*. Also, a large number of mesophyll cells on the adaxial/abaxial surface of *sdl* lateral veins disappeared, these defects directly caused the drooping leaf (Qin et al. 2017). Since RNR plays an important role in the *de novo* synthesis of dNTP, the relative dNTP levels decreased in the same trend in *st1* and *nsl2*. But it decreased even more in the *nsl2* mutant. This may suggest that *st1* and *nsl2* is weak and strong mutation, respectively. Collectively, all the three allele mutant *nsl2*, *st1*, *sdl* exhibited stripe leaf. Owning to their different mutation forms and different mutation sites, some new phenotypes have emerged. The *sdl* mutant also displayed additional drooping leaf, dwarfism and the abnormal floral organs. Interestingly, in the current study, *nsl2* showed narrow leaf and the molecular mechanism remains unclear. Consequently, the allelic gene *NSL2* of *ST1* and *SDL* has a new function in regulating leaf width.

### New Insight into the Functions of Ribonucleotide Reductase (RNR) Protein

It is known that ribonucleotide reductase (RNR) is the rate-limiting enzyme for the synthesis of deoxyribonucleic acid, which reduces ribonucleic acid to deoxyribonucleic acid, plays a pivotal role in the DNA synthesis and repair in all living organisms (Guarino et al. 2014). RNR is a polymer formed by the combination of large and small subunits (RNRL/RNRS). The majority of knowedge about the activity and regulation of RNR has come from studies in Escherichia coli, yeast and mouse (Elledge and Davis 1990; Jordan and Reichard 1998). It has been reported that three RNRS genes in Arabidopsis, *TSO2*, *RNR2A* and *RNR2B*, displayed a degree of functional redundancy, and a severe developmental defect phenotype was observed in the double mutant (Wang and Liu 2006). *SvSTL1* encodes the large subunit of RNRs in the *S.viridis*, and *svstl1* showed bleached flag leaves and disrupted chloroplast development (Li et al. 2022). *V3* and *ST1* encode the RNRL1 and RNRS1, respectively, which are required for chloroplast biogenesis in rice. The *v3* and *st1* mutants showed chlorotic leaves in a growth stage-dependent manner under field conditions (Yoo et al. 2009). The *sdl* mutant showed stripe and drooping leaf, dwarfism and the abnormal floral organs (Qin et al. 2017). In our present study, it was found that *nsl2* exhibited not only stripe leaf, but also present narrow leaf, which provided a new insight into the functional study of RNRS1.

### ***NSL2*** Might Control Leaf Width by Affecting Cell Cycle Progression

Leaf morphology is an important factor for creating the ideal plant architecture, having a crucial influence on rice yield. However, most of the studies on the molecular mechanism of leaf development up to today focused on dicots, especially *Arabidopsis thaliana*. So it has important theoretical significance and application value to study the molecular mechanism of leaf development in rice, the model plant of monocot. Leaf development involves a complexed process that initiates from the SAM. The morphogenesis of the leaf is determined by the leaf founder cells on the longitudinal axis, mediolateral axis and dorsal-ventral axis of the SAM periphery. The shape and size of the leaf are determined by the coordinated regulation of cell division and expansion along these axes (Moon and Hake 2011; Gonzalez et al. 2012). Leaf width is an important component of leaf morphology, and a moderate leaf shape is conducive to maintaining its upright gesture, improving photosynthetic efficiency and increasing rice grain production (Tsukaya 2006; Micol 2009). In this study, no significant difference in cell width was observed between WT and *nsl2* mutant, thus we speculated that *NSL2* might control leaf width by affecting cell cycle progression.

**Fig. 6:**
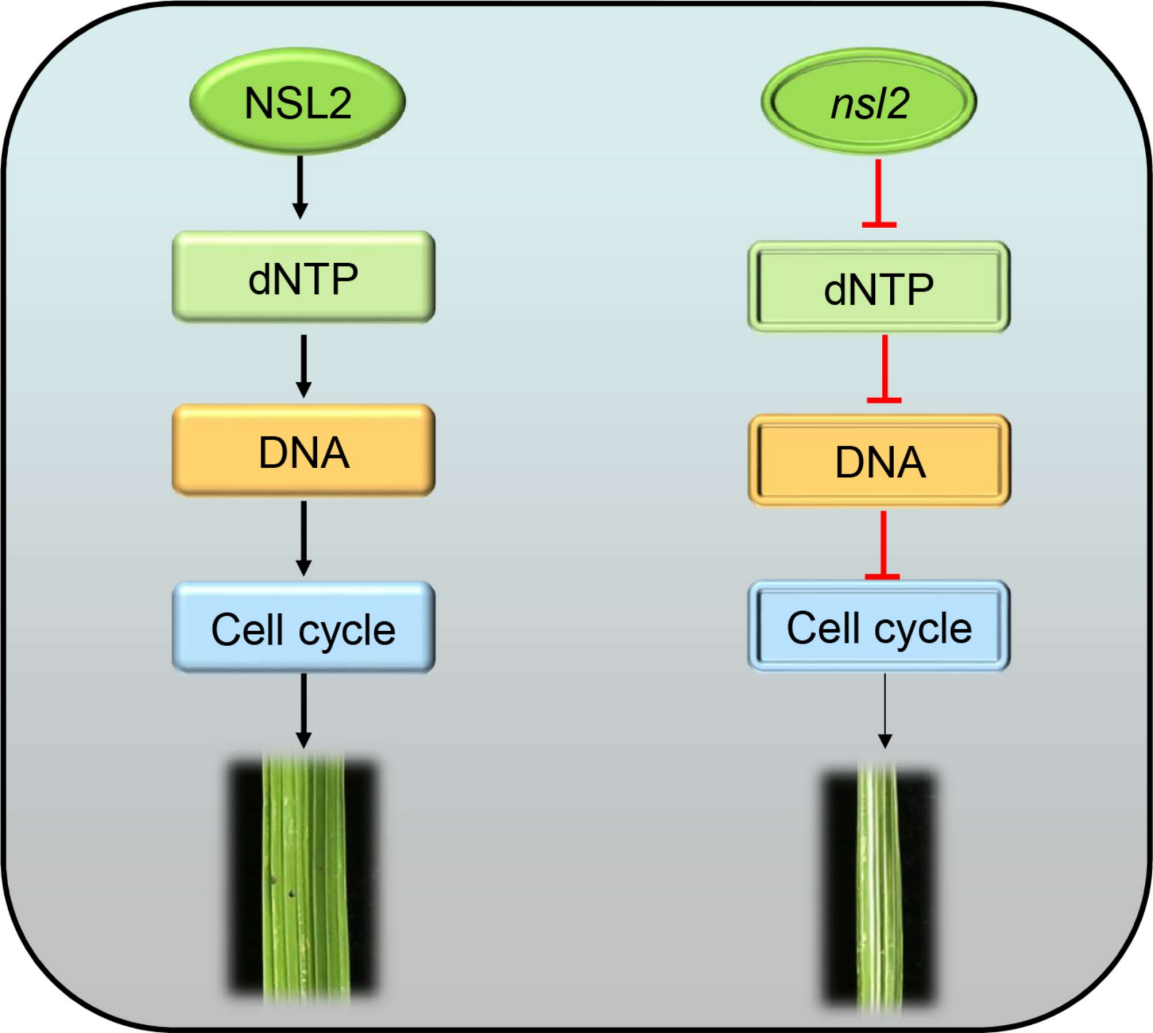
Proposed model for *NSL2* regulate leaf width

To date, several genes affecting leaf width in rice had been identified, including the WUSCHEL-related homeobox (WOX) genes *NAL2* and *NAL3*, the SHAQKYF class MYB transcription factor *SLL1*, the micro-RNA related gene *DCL1*, the cellulose synthase-like protein D4 gene *NRL1*, the ribosomal small subunit protein RPS3A gene *NAL21*, and the allelic genes *AVB*, *SRL2*, *NRL2* (Liu et al. 2005; Guang-Heng et al., 2009; Hu et al., 2010; Ishiwata et al., 2013; Liu et al., 2016; Zhao et al., 2016; Ma et al., 2017; Uzair et al., 2021). Here, we identified a novel narrow leaf mutant which had a decrease in the distances between the small vascular bundles, and the numbers of small and large vascular bundles, compared with the WT. Furthermore, *nsl2* was deficient in dNTPs which is needed in DNA duplication. Therefore, the deficiency of dNTPs impedes DNA synthesis and leads to a reduced rate of cell proliferation (Fig. 6). Therefore, we concluded that *NSL2* plays a critical role in leaf width by affecting cell cycle progression in rice.

### Potential Application of *NSL2* in Rice Breeding

Rice is not only a monocot model plant, but also an important food crop. A higher yield has always been a primary goal pursued by rice breeders. The shaping of ideal plant architecture of rice is an important way to improve rice yield. Since leaf is the main organ for photosynthesis, leaf width is a key component of plant architecture. Appropriate leaf width must have important biological significance for improving light absorption and light-energy-to-chemical energy conversion efficiency (Ort et al. 2015). In this study, *NSL2* which associated with leaf width was characterized, the width of the flag leaf, last second and last third leaf were reduced by 0.32, 0.39 and 0.59 cm in the *nsl2* mutant. In addition, *nsl2* showed stripe leaf. However, with no additional influence on leaf length, leaf number, leaf angle, and thus is easy to use. Therefore, *NSL2* gene could be utilized in rice breeding.

## Conclusions

A novel allelic *nsl2* mutant was characterized in rice and showed narrow and strip leaf. The *NSL2* encodes a small subunit of ribonucleotide reductase, which was a nucleus and cytoplasm protein and highly expressed in leaf. Polymerase-based assay showed that *NSL2* decreases dNTP level in *nsl2* mutant. Flow cytometric analysis and the altered transcript level of genes related to cell cycle confirmed that cell cycle progression was impaired in *nsl2* mutant. Taken together, our results indicated that *NSL2* play a crucial role in regulating leaf width by modulating cell cycle progression in rice. Furthermore, this study provide a new insight into the function ribonucleotide reductase in plant.

## Materials and methods

### Plant Materials and Growth Conditions

The rice (*Oryza sativa*) mutant *nsl2* showing a characteristic phenotype of narrow leaves was derived from an ethyl methane sulfonate (EMS) mutagenesis of an *indica* rice cultivar Jinhui10 (J10). J10 was used as the wild type (WT) in all experiments.

Rice plants were cultivated in the experimental field at the Rice Research Institute of Southwest University, Chongqing, China under natural growing conditions.

### Morphological and Histological Analysis

Leaves were taken from *nsl2* and wild-type plants was examined using a scanning electron microscope (SU3500; Hitachi) with a -20 °C cool stage under a low-vacuum environment.

Fresh leaves were collected and fixed in FAA (3.7% [v/v] formaldehyde, 50% [v/v] alcohol and 0.9 M [m/v] glacial acetic acid) for 16–24 h at 4 °C after vacuum infiltration. The fixed leaves were dehydrated with a graded alcohol series, infiltrated with xylene, and embedded in paraffin. Thin Sect. (8 μm thick) were prepared with a microtome (RM2245; Leica), then deparaffinized in xylene, and dehydrated through an alcohol series. The sections stained with 1% safranin (Amresco Inc., Framingham, MA, USA) and 1% fast green (Amresco Inc., Framingham, MA, USA), and a coverslip was mounted with neutral balsam. The sections were observed using an E600 microscope (Nikon).

### Epidermal Cell Observations

The third-leaf blades of *nsl2* and wild-type plants were fixed with formaldehyde: glacial acetic acid:50% ethanol (2:1:17) for 24 h at 4 °C. They were then dehydrated in a graded ethanol series.

Dehydrated samples were incubated at 96 °C in chloral hydrate dissolved in 100% ethanol until they cleared and were then observed under a light microscope.

### Map-Based Cloning of *NSL2*

The F_1_ hybrids between Xinong 1 A and *nsl2* were constructed and planted. A total of 1,500 recessive mapping plants from the F_2_ population were used for fine mapping and isolation of the *NSL2* gene. Based on the findings in Sang et al. (2010), two polymorphic insertion/deletion (InDel) markers Ind6-1 and Ind6-2 were developed in the 345 kb region between simple sequence repeat (SSR) marker RM19746 and RM19762. Candidate genes were predicted using the Gramene database (http://www.gramene.org/) and the Rice Genome Annotation Project (http://rice.plantbiology.msu.edu). The sequences of primers used in the mapping and candidate gene analysis are listed in Supplemental Table S1.

### Vector Construction and Transformation

For functional complementation test, a 3,919-bp genomic fragment that contained the 2,000-bp upstream sequence, 1,020-bp coding sequence, and 899-bp downstream sequence, was amplified using *NSL2*-com-F and *NSL2*-com-R from the WT genomic DNA. The fragment was digested using *Kpn*I and *Xba*I, and inserted into the binary vector pCAMBIA1301 by pEASY-Uni Seamless Cloning and Assembly Kit (Transgene). The *NSL2* complementation recombinant plasmid was introduced into the *nsl2* mutant by *Agrobacterium*-mediated transformation as described previously (Zhang et al. 2017). The sequences of primers used in the vector construction are listed in Supplemental Table S1.

### Subcellular Localization

In order to confirm the subcellular localization of NSL2 protein, the *NSL2* coding sequence without the stop codon was amplified from WT using the primers designated pAN580-NSL2-F and pAN580-NSL2-R. The fragment was cloned into the transient expression vector pAN580 at the *Xbal*I/*Bam*HI site to generate NSL2-GFP construct. After verification by sequence analysis, the pAN580-GFP and NSL2-GFP plasmids were transformed into rice protoplasts by polyethylene glycol-mediated method. After incubation at 28ºC for 12–16 h, fluorescence was observed using a confocal laser scanning microscope (LSM800, Zeiss, Jena, Germany). The primers are shown in Table S1.

### Protein Sequence and Phylogenetic Analysis

The NSL2-related protein sequences were identified from Phytozome (https://phytozome-next.jgi.doe.gov/) using the full-length amino acid sequence of NSL2 as a query. A phylogenetic tree was constructed using the maximum likelihood method based on the Jones-Taylor-Thornton matrix-based model with the lowest Bayesian information criterion scores by MEGA 5.0 (Tamura et al. 2011). Statistical support for the tree topology was assessed by means of a bootstrap analysis with 500 replicates.

### Measuring dNTP Levels

The dNTP levels were measured by a polymerase-based assay as described previously (Roy et al. 1999; Wang and Liu 2006). In brief, samples of the WT and *nsl2* mutant were harvested and ground to a fine powder in liquid nitrogen. Two hundred milligrams of ground tissues were violently vortexed with 60% ice-cold methanol, followed by an incubation at 95℃ for 5 min, and centrifuged at 17,000 g for 20 min. The supernatant was dried in a SpeedVac (Hanil), resuspended in 0.1 mL sterile distilled water and stored at -20℃. Commercial dNTPs (Promega) were used to generate a linear standard curve.

### RNA Isolation and RT-qPCR Analysis

Total RNA was extracted from various tissues using the RNAprep Pure Plant Kit (Tiangen) following the manufacturer’s instructions. The first-strand cDNA was synthesized based on the PrimeScript®Reagent Kit with gDNA Eraser Kit (Takara, Dalian, China). qPCR was performed on a CFX96 Connect™ Real-time System (Bio-Rad, Hercules, CA, United States) using the SYBR Premix Ex Taq II Kit (Takara) with rice Actin as the endogenous control. At least three biological eplicates were performed. All primer pairs used for RT-qPCR are listed in Supplemental Table 1.

### Flow Cytometric Analysis

For nuclear staining, the shoot apex from tilling-stage rice was cut into small pieces in 400 µL of extraction buffer (Partec CyStain UV Precise P) on ice, and then filtered with a 30-µm CellTtics filter to remove cellular debris. After staining with Partec CyStain UV Precise P Staining solution (including DAPI) (Sysmex Partec), the nuclei were analyzed using CyFlow Space (Sysmex Partec). A total of 30,000 events were recorded, and the data were analyzed using the FCS Express software (https://www.denovosoftware.com/).

## Electronic Supplementary Material

Below is the link to the electronic supplementary material.

**Additional file 1: Fig. S1** Leaf blade length of the WT and *nsl2* plants. Data presented are the means ±SD from ten independent plants. The P-values were determined using Student?s test. *P<0.05, **P<0.01. **Fig. S2** Scanning electron micrographs of leaf of the WT (a) and *nsl2* mutant (b). Bule arrow and white arrow represents the large vascular bundles (LV) and small vascular bundles (SV), respectively. Bars (a and b) 500 ?m. **Fig. S3** Chlorophyll (Chl) content of leaves in WT and *nsl2* plants. FW, fresh weight. Data presented are the means ±SD from three independent experiments. The P-values were determined using Student’s test. *P<0.05, **P<0.01. **Fig. S4** Sequence peak chromatograms of the mutation region in plants of the WT, *nsl2*, and *nsl2*-com. **Table S1** Primers used in this study. **Table S2** The important agronomic traits of WT and *nsl2* mutant. **Table S3** ORFs in the narrowed region.

## Data Availability

All data generated or analysed during this study are included in this published article and its supplementary information files.

## Abbreviations

WT: Wild-type
*NSL2*: *NARROW AND STRIPE LEAF 2*
*nsl2*: *Narrow and stripe leaf 2*
SAM: Shoot apical meristem
RNR: Ribonucleotide reductase
EMS: Ethyl methane sulfonate
CTAB: Cetyltrimethylammonium bromide
dNTP: Deoxy-ribonucleoside triphosphate
GFP: Green fluorescent protein
qPCR: Quantitative PCR
SEM: Scanning electron microscopy
LVs: Large vascular bundles
SVs: Small vascular bundles.
