# Supplementary material for: Narrow and Stripe Leaf 2 Regulates Leaf Width by Modulating Cell Cycle Progression in Rice
Source: Rice (N Y). 2023 Apr 18;16:20. doi: 10.1186/s12284-023-00634-3 (PMC10113404; doi:10.1186/s12284-023-00634-3)
Supplement: Supplementary file 1 — Additional file 1: Fig. S1 Leaf blade length of the WT and nsl2 plants. Data presented are the means ±SD from ten independent plants. The P-values were determined using Student?s test. *P<0.05, **P<0.01. Fig. S2 Scanning electron micrographs of leaf of the WT (a) and nsl2 mutant (b). Bule arrow and white arrow represents the large vascular bundles (LV) and small vascular bundles (SV), respectively. Bars (a and b) 500 ?m. Fig. S3 Chlorophyll (Chl) content of leaves in WT and nsl2 plants. FW, fresh weight. Data presented are the means ±SD from three independent experiments. The P-values were determined using Student’s test. *P<0.05, **P<0.01. Fig. S4 Sequence peak chromatograms of the mutation region in plants of the WT, nsl2, and nsl2-com. Table S1 Primers used in this study. Table S2 The important agronomic traits of WT and nsl2 mutant. Table S3 ORFs in the narrowed region. [file 12284_2023_634_MOESM1_ESM.docx]

**Supplementary Information**

Additional Supplementary data may be found online in the Supplementary data tab for this article.

**Additional file 1: Fig. S1** Leaf blade length of the WT and *nsl2* plants. Data presented are the means ± SD from ten independent plants. The P-values were determined using Student’s test. *P<0.05, **P<0.01.

**Additional file 2: Fig. S2** Scanning electron micrographs of leaf of the WT (a) and *nsl2* mutant (b). Bule arrow and white arrow represents the large vascular bundles (LV) and small vascular bundles (SV), respectively. Bars (a and b) 500 µm.

**Additional file 3: Fig. S3** Chlorophyll (Chl) content of leaves in WT mutant and *nsl2* plants. FW, fresh weight. Data presented are the means ± SD from three independent experiments. The P-values were determined using Student’s test. *P<0.05, **P<0.01.

**Additional file 4: Fig. S4** Sequence peak chromatograms of the mutation region in plants of the WT, *nsl2*, and *nsl2*-com.

**Additional file 5: Table S1** Primers used in this study.

**Additional file 6: Table S2** The important agronomic traits of WT and *nsl2* mutant.

**Additional file 7: Table S3** ORFs in the narrowed region.

**Additional file 1: Fig. S1**

**
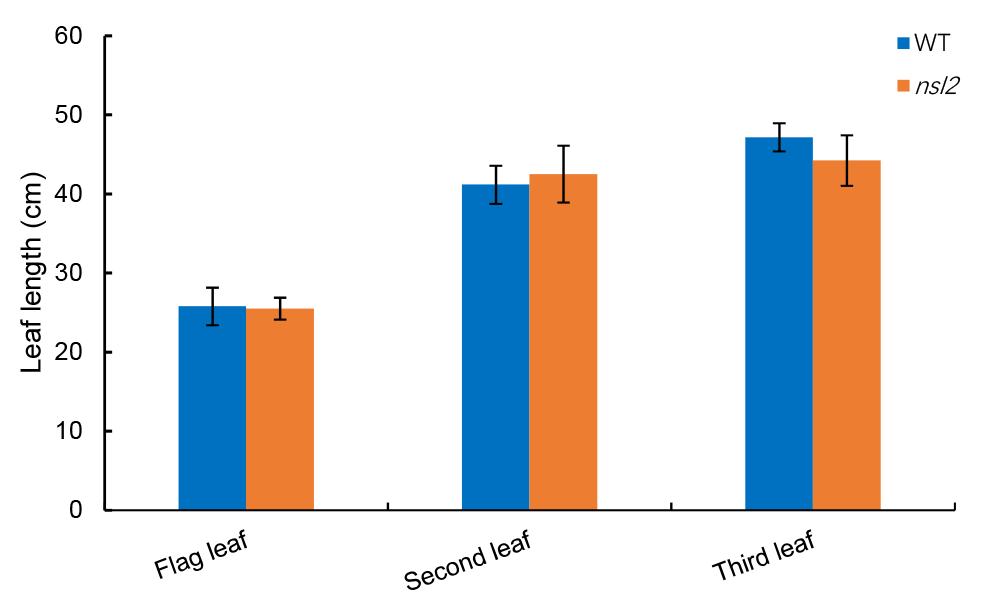
**

**Additional file 2: Fig. S2**

**
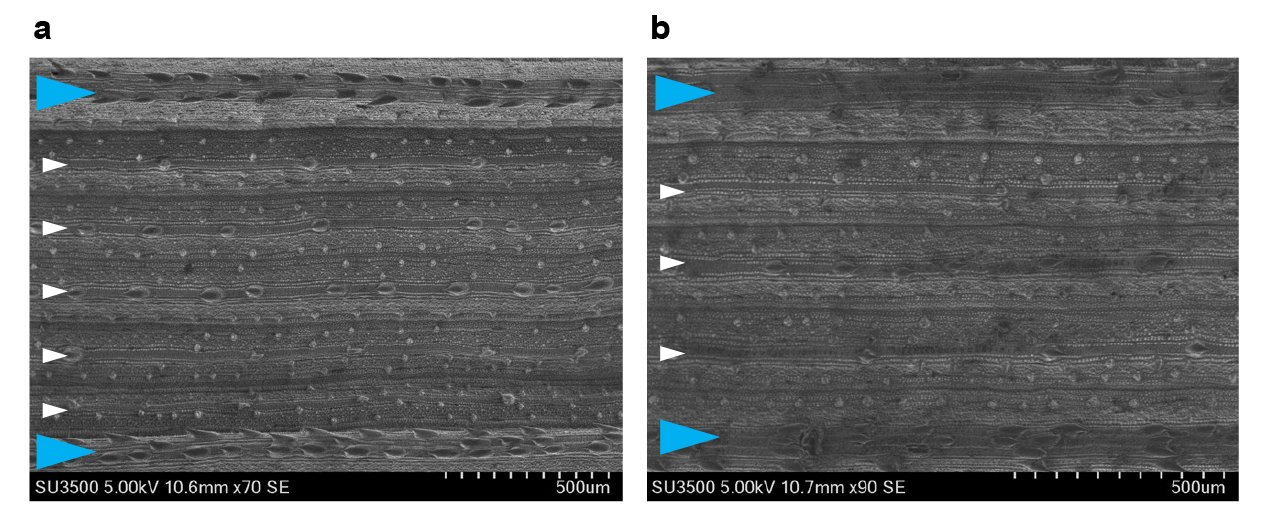
**

**Additional file 3: Fig. S3**

**
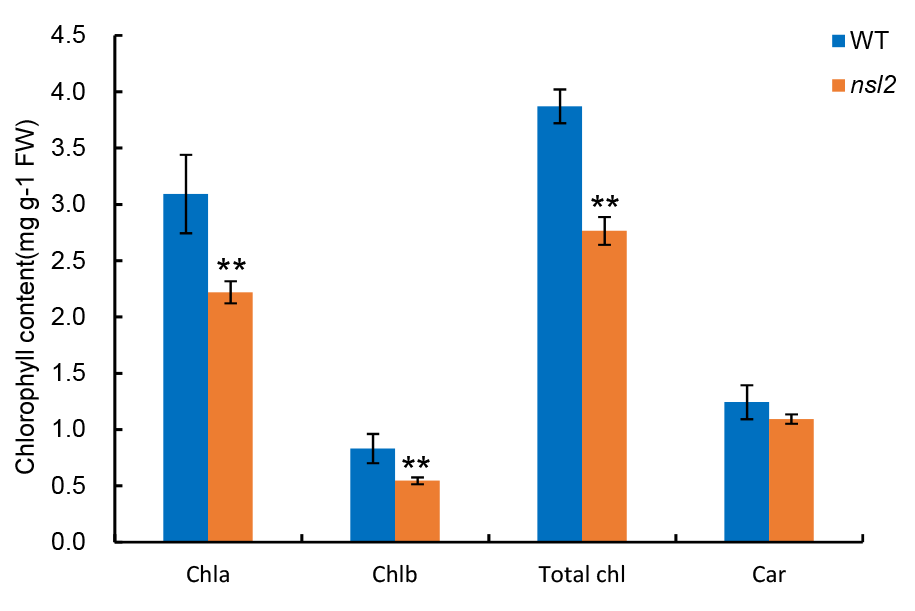
**

**Additional file 4: Fig. S4**

**
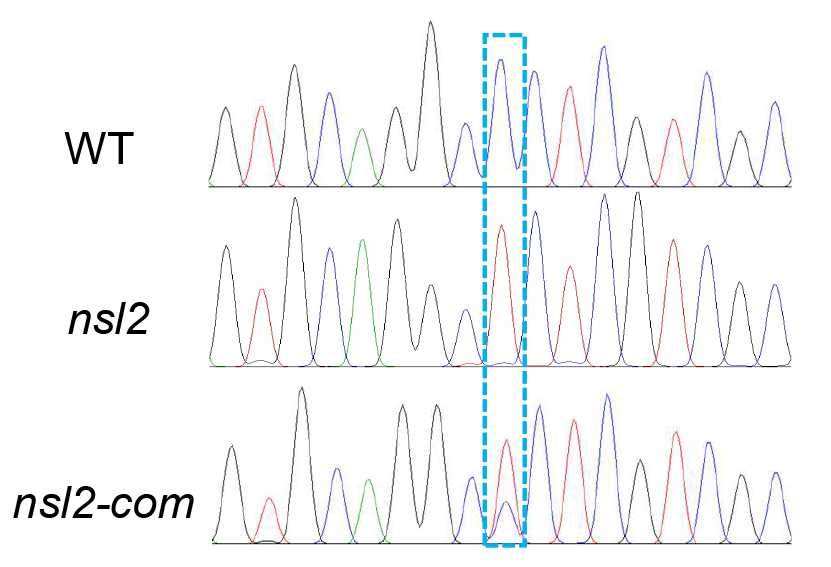
**

**Table S1. Primers used in this study**

| Use | Primer name | Sequence(5’to 3’) |
| --- | --- | --- |
| Gene mapping | Ind6-1-F | CCGGTTAGATATCAGATCCCG |
|  | Ind6-1-R | CCTCGCAAAATCACCTGACACGTG |
|  | Ind6-2-F | GTAACCATATAGGACTAG |
|  | Ind6-2-R | CTCCTATGTCGGATGGCTTGC |
| Binary vector construction | *NSL2*-com-F | ACAGCTATGACATGATTACGAATTCCCATGTCATCTGAAATTGCTCTCAA |
|  | *NSL2*-com-R | TGCATGCCTGCAGGTCGACTCTAGAGAGGACTAGAGGAGCGTCAGTGGGC |
| Subcellular localization | pAN580- NSL2-F | AGGACAGCCCAGATCAACTAGTATGCCGGCCGCGCCGACG |
|  | pAN580- NSL2-R | GGTCCTCGAGACGTCTCTAGAGAAGTCTTCGTCGATGCTGAAGACGTG |
| qRT-PCR | NSL2-F | GCGCCTTCTACGGATTCCAGATC |
|  | NSL2-R | CTCGGCGAAGCGCTCGCC |
|  | R2-F | TCTGCACCTCCACTTCGCTCA |
|  | R2-R | TAGGTGGTGGCCTTGGAAGCT |
|  | H4-F | GCAAGTACCAGAAGAGCACG |
|  | H4-R | AGAGGTTGGTGTCCTCGAAG |
|  | CAK1A-F | GACCGACAAGGGTTTCAGCAT |
|  | CAK1A-R | CCAGCATGTTCAGGAAGATACAAT |
|  | CDKA;1-F | GGTTTGGACCTTCTCTCTAAAATGC |
|  | CDKA;1-R | AGAGCCTGTCTAGCTGTGATCCTT |
|  | CDKA;2-F | CGAGATTTGAAGCCCCAGAA |
|  | CDKA;2-R | TCCGCGAGCTTCAATGAGTT |
|  | CYCD4-F | GCCATGGAGTTGATACATCCAA |
|  | CYCD4-R | CCAGTAGGGCTCCGTGGAAT |
|  | E2F2-F | TGTTGGTGGCTGCCGATAT |
|  | E2F2-R | CGCCAGGTGCACCCTTT |
|  | KRP5-F | GCGACAACGTTCTTGACCTC |
|  | KRP5-R | AGGCGTTGTCTCCCTGGT |
|  | RB1-F | CTCCTATCCAGCATGTCATCCT |
|  | RB1-R | AGCCTTGACACACAGTAAAGA |
| Internal control | ACTIN-F | TGCTATGTACGTCGCCATCCAG |
|  | ACTIN-R | AATGAGTAACCACGCTCCGTCA |

**Table S2. The important agronomic traits of WT and *nsl2* mutant.**

| **Agronomic trait** | **WT** | ***nsl2*** |
| --- | --- | --- |
| Plant height (cm) | 110.52±1.18 | 98.78±2.23** |
| No. of effective panicle | 10.40±2.19 | 10.30±2.01 |
| No. of primary branches per panicle | 11.80±0.75 | 9.50±0.50** |
| No. of secondary branches per panicle | 41.60±6.05 | 20.63±3.19** |
| No. of spikelets per panicle | 170.40±22.04 | 111.37±10.18** |
| Seed setting rate (%) | 81.89±3.11 | 62.92±3.57** |
| 1,000‐grain weight(g) | 21.61±0.25 | 22.12±0.32 |

**Table S3** ORFs in the narrowed region

| **Gene** | **Gene Product Name** |
| --- | --- |
| *LOC_Os06g14560* | expressed protein |
| *LOC_Os06g14570* | retrotransposon protein, putative, Ty3-gypsy subclass, expressed |
| *LOC_Os06g14580* | hypothetical protein |
| *LOC_Os06g14590* | retrotransposon protein, putative, Ty3-gypsy subclass, expressed |
| *LOC_Os06g14600* | retrotransposon protein, putative, Ty3-gypsy subclass, expressed |
| *LOC_Os06g14610* | retrotransposon protein, putative, Ty3-gypsy subclass, expressed |
| *LOC_Os06g14620* | **small subunits of ribonucleotide reductase** |
| *LOC_Os06g14630* | GDSL-like lipase/acylhydrolase, putative, expressed |
| *LOC_Os06g14640* | zinc finger, C3HC4 type domain containing protein, expressed |
| *LOC_Os06g14650* | zinc finger, C3HC4 type domain containing protein, expressed |
